# A Systems Approach to Endophyte-Mediated Plant Holobiont and Microbiome Dynamics

**DOI:** 10.3390/plants15050802

**Published:** 2026-03-05

**Authors:** Deepak Kumar, Krishna Sundari Sattiraju, M. S. Reddy

**Affiliations:** 1 Department of Biotechnology, Jaypee Institute of Information Technology, A-10, Sector-62, Noida 201309, Uttar Pradesh, India; 2404010021@mail.jiit.ac.in; 2 Asian PGPR Society for Sustainable Agriculture, Auburn University, Auburn, AL 36849, USA; prof.m.s.reddy@gmail.com

**Keywords:** endophytes, stress tolerance, multi-omics, holobiont, CRISPR, systems biology, plant–microbe interactions

## Abstract

The plant holobiont comprises the host plant and its associated microbial communities functioning together as a single ecological and evolutionary unit that influences plant health, productivity, and environmental adaptability. Endophytes, formerly classified primarily as plant growth-promoting agents, are currently gaining traction as integral components of plant-associated microbiomes such as the rhizobiome and phytobiome. They can alter host-mediated root exudation patterns, microbial community structure, and nutrient dynamics within the rhizosphere. Endophytes play an important role in modulating host signaling pathways, thus influencing plant growth. Various mechanisms by which endophytes contribute to improved plant performance include soil microbiome dynamics, carbon sequestration, and strengthening the host’s ability to tolerate abiotic stressors. Multi-omics, single-cell, and systems-level approaches integrated with CRISPR, metabolic engineering, and AI, together with systems biology, guided by in vitro and field studies, support predictive modeling and provide evidence for the evolution of system-driven strategies for developing effective bioinoculants. This review highlights the potential of endophytes to serve as a scalable and sustainable component of climate-resilient and regenerative agricultural systems, while acknowledging ecological variability and field-level constraints.

## 1. Introduction

**Figure 1:**
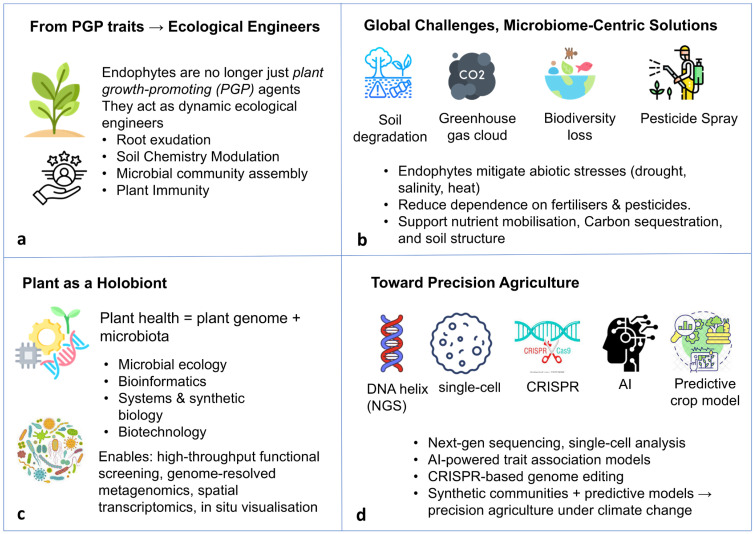
Conceptual framework illustrating the evolving role of endophytes within the plant holobiont and their application in sustainable agriculture. (**a**) Shift from plant growth-promoting agents to ecological engineers influencing root exudation, soil chemistry, microbial assembly, and immunity. (**b**) Microbiome-based solutions to global challenges through stress mitigation, reduced agrochemical use, and improved nutrient cycling and soil health. (**c**) Plant holobiont concept integrating plant genome and microbiota, supported by advances in systems biology and biotechnology. (**d**) Precision agriculture enabled by next-generation sequencing, single-cell tools, CRISPR, AI, and predictive crop models.

Over the past decade, the paradigm of endophytic interactions within the broader plant–microbe interface has undergone a fundamental transformation. Endophytes, once identified as plant growth-promoting microorganisms (PGP), are now gaining traction as dynamic and multifunctional partners that play pivotal roles in both the structural and functional modulation of the rhizobiome and phytobiome. This shift in perspective reflects an expanded ecological understanding of endophytes as integral components of plant-associated microbiomes, rather than implying repeated or distinct conceptual reclassifications [1,2,3]. Endophytes function as integral components of the plant holobiont, influencing plant–soil–microbiome interactions and enabling microbiome-centric strategies for sustainable agriculture (Figure 1). Endophytes exert their influence through diverse mechanisms, including the modulation of root exudation profiles, plant-mediated alteration of soil physicochemical properties, the orchestration of microbial community assembly, and fine-tuning of plant immune responses via intricate molecular signaling networks [2]. Changes in soil nutrient availability, aggregation, and microbial activity associated with endophyte colonization are primarily indirect and arise through modifications of plant physiology and root-derived inputs, rather than direct physicochemical transformation of soil matrices [2,3,4,5]. Such functions underscore the central role of endophytes in determining plant performance under both optimal and stress-prone conditions. The urgency to harness endophyte-mediated solutions is heightened by escalating environmental challenges, including severe soil degradation, loss of soil biodiversity, depletion of organic matter, advancing desertification, increased greenhouse gas emissions, and climate-driven disruptions to agroecosystem productivity [6]. In this context, microbiome-centric strategies represent a promising and sustainable alternative to conventional chemical-intensive agricultural practices. Endophytes are reported to possess diverse biological traits that enhance plant tolerance to abiotic stresses such as drought, salinity, and temperature extremes. Simultaneously, researchers observed reduced dependency on synthetic fertilizers and pesticides in the presence of endophytes [7]. Beyond stress mitigation, endophytes play critical roles in nutrient mobilization, indirect carbon stabilization via enhanced plant productivity and soil organic matter inputs, soil aggregation, and overall soil structural stability, positioning them as key contributors to sustainable land-use practices and regenerative agriculture at scale [8]. This expanded functional understanding aligns with the broader microbiome revolution, which recognizes that plant health and productivity are determined not solely by the plant genome but by the collective genetic and functional attributes of the plant-associated microbiota—the holobiont [9,10,11].

The review aims to synthesize current knowledge on endophyte-mediated mechanisms regulating plant performance, microbiome assembly, and stress resilience, with a focus on molecular, ecological, and applied perspectives. The scope of the review is limited to experimentally supported interactions involving bacterial and fungal endophytes in agricultural and natural systems. It further organizes advances in mechanistic insights and how they drive translational developments through the incorporation of emerging technologies and provides a structured framework. Realizing the full potential of endophytes necessitates a rigorously interdisciplinary framework integrating microbial ecology, molecular biology, bioinformatics, systems biology, synthetic biology, and biotechnology. Specifically, next-generation sequencing and genome-resolved metagenomics are discussed for their roles in profiling endophyte diversity and functional potential, while single-cell and spatial transcriptomic approaches are highlighted for resolving host–endophyte interactions at cellular resolution. Artificial intelligence (AI)-driven modeling is included for trait prediction and consortium design, and clustered regularly interspaced short palindromic repeats (CRISPR)-based genome editing is discussed as an emerging tool for functional validation rather than routine application. These complementary approaches facilitate a mechanistic understanding of how endophytes influence host physiology and ecosystem processes [12,13,14]. Rapid technological advancements are accelerating the discovery, characterization, and targeted deployment of elite endophytic strains tailored to specific crops and environments [14]. Building upon this conceptual foundation, a systems-level perspective is required to understand how endophytes function as regulators of plant–microbiome systems.

## 2. Systems View of Endophytic Bacteria: From Symbionts to Rhizobiome Engineers

Adopting a systems-level perspective on endophytic bacteria represents a significant conceptual advance in our understanding of plant–microbe interactions. Traditionally regarded as passive inhabitants or, at best, plant growth-promoting symbionts, endophytes are now receiving increasing attention as active ecological engineers that shape the structure and function of the rhizobiome [15]. This paradigm shift reflects a transition from descriptive, organism-centric views toward a functional and interaction-based framework for understanding microbiome-mediated plant processes. Central to this reorientation is the move away from strict taxonomic classification toward function-driven interpretations of endophytic activity. Rather than prioritizing phylogenetic identity, contemporary approaches emphasize the ecological roles performed by endophytes—such as nutrient cycling, stress mitigation, and immune modulation—which are more directly linked to plant fitness and ecosystem performance [16,17]. Functional redundancy is a defining characteristic of endophytic communities, wherein multiple taxonomically distinct microorganisms perform similar beneficial roles, including nitrogen fixation, phytohormone modulation, and phosphate solubilization. This redundancy enhances ecosystem resilience by ensuring functional continuity under fluctuating environmental conditions [18]. At the plant–soil interface, these functional attributes manifest through modifications in root system architecture, alterations in root exudation profiles, and shifts in soil carbon dynamics. Collectively, these processes influence the composition, activity, and assembly of the surrounding microbial community, reinforcing the role of endophytes as drivers of rhizobiome organization rather than passive residents [19]. Such feedback mechanisms exemplify how endophytes integrate plant physiological responses with broader soil microbial networks.

Endophytes also exhibit remarkable ecological plasticity, dynamically transitioning among commensal, mutualistic, or opportunistic lifestyles depending on host genotype, developmental stage, and environmental stressors. This context-dependent behavior underscores their adaptability and highlights their potential as targeted bio-intervention agents in agroecosystems exposed to biotic and abiotic pressures [20,21]. The emergence of the core–satellite microbiome concept has further refined our understanding of endophytic community organization. Within this framework, a relatively small subset of consistently enriched bacterial taxa often referred to as functional guilds or microbial keystones exerts disproportionate influence on plant health and productivity, irrespective of soil type or geographic location [22,23]. These keystone endophytes contribute to microbiome stability, suppression of pathogens, modulation of host gene expression, and maintenance of metabolic balance, thereby playing a central role in holobiont functionality [24]. Together, these insights position endophytic bacteria not merely as supportive symbionts, but as integral system-level regulators of plant–microbiome dynamics, with profound implications for sustainable agriculture, ecosystem resilience, and microbiome engineering strategies.

## 3. Endophyte-Driven Modulation of Plant–Microbiome Networks

### 3.1. Rhizobiome and Holobiome Reprogramming

Endophyte-mediated regulation of plant–microbiome networks, particularly through rhizobiome and holobiome reprogramming, represents a highly dynamic and adaptive process involving chemical signaling, microbial community restructuring, and multi-level ecological feedback. Central to this process is the endophyte-induced modulation of root exudation, which serves as a primary interface for communication between the host plant and its associated microbial communities [25,26]. Colonization by endophytes can substantially alter both the concentration and composition of root exudates, thereby reshaping microbial recruitment and activity in the rhizosphere. For instance, the endophytic fungus *Phomopsis liquidambaris* B3 was shown to significantly modify root exudate profiles in peanut plants, facilitating enhanced chemotaxis, growth, and biofilm formation of symbiotic rhizobia. Authors reported elevated levels of organic acids (e.g., citric and oxalic acids), phenolic compounds (e.g., cinnamic and ferulic acids), flavonoids (e.g., quercetin and biochanin A), amino acids (e.g., glutamate, glycine, and glutamine), and other metabolites known to play critical roles in microbial attraction and signaling [25,27]. These exudate-driven changes might exert direct bioactive effects on rhizobial behavior, including substantial upregulation of nodulation-associated genes such as *nodC*, resulting in increases up to 169.2% relative to non-inoculated controls under in vivo conditions [25]. Beyond rhizobial recruitment, altered exudation patterns may function as selective filters that preferentially attract beneficial microorganisms while suppressing opportunistic or pathogenic taxa, thereby facilitating the assembly of a functionally advantageous rhizobiome [26].

This community reorganization is further reinforced by sophisticated biochemical interactions, wherein endophyte-derived signaling molecules act as regulators of microbial gene expression, motility, aggregation, and biofilm formation—processes central to community stability and function [28]. In parallel, allelopathic interactions and metabolic cross-feeding contribute to microbiome stabilization by selectively inhibiting competing microorganisms while promoting cooperative metabolic networks among beneficial taxa. Evidence from intercropping systems further supports the role of endophytes in mediating allelochemical interactions that enable plants to actively engineer their associated microbial communities. Through endophyte-facilitated modulation of allelochemical release, plants can bias microbiome composition toward configurations that enhance nutrient acquisition, stress tolerance, and pathogen suppression [29]. Collectively, these mechanisms illustrate how endophytes function as central orchestrators of rhizobiome and holobiome reprogramming, enabling plants to dynamically optimize microbial partnerships in response to environmental and developmental cues. Beyond rhizobiome restructuring, endophytes also influence higher trophic ecological interactions.

### 3.2. Multi-Trophic Interactions

Multi-trophic interactions involving endophytes and other plant-associated organisms, including mycorrhizal fungi, protists, nematodes, and pathogens, constitute a complex and dynamic ecological network that collectively shapes plant health, resilience, and functional adaptability. Although the importance of these interactions is gaining prominence, many aspects of their interdependence and regulatory mechanisms remain poorly understood [30]. Endophytes do not merely coexist within this network; rather, they actively influence both the direction and outcome of interactions among multiple trophic levels within the plant microbiome. At the molecular level, these interactions are mediated by conserved plant signaling pathways, including mitogen-activated protein kinase (MAPK) cascades that integrate microbial perception with downstream hormonal responses (MPK3/MPK6-dependent signaling) [31].

Endophytes were shown to exert substantial control over mycorrhizal symbioses, particularly arbuscular mycorrhizal fungi (AMF). Several endophytic fungi and bacteria, including *Piriformospora indica* and *Epichloë* species, are known to modulate AMF colonization dynamics and symbiotic efficiency [32,33]. Experiments by Daneshkhah et al. (2018) indicated the requirement of host MPK6 activity to establish stable colonization and coordinated jasmonic acid (JA)- and ethylene (ET)-dependent transcriptional reprogramming during symbiosis formation in *Piriformospora indica* [34]. The mechanistic basis of synergistic or antagonistic AMF endophyte interactions is governed by host-regulated carbon allocation, root exudate-mediated microbial gating, and phytohormonal prioritization. Both AMF and endophytes function as carbon sinks dependent on host-derived photosynthates; however, plants appear to regulate carbon partitioning by preferentially allocating resources to symbionts that confer greater functional benefits under specific environmental conditions [35,36].

Depending on species composition and environmental context, endophytes may either inhibit mycorrhizal colonization through competitive exclusion or the production of antimicrobial metabolites or promote AMF establishment by enhancing phosphorus solubilization and modifying root exudate chemistry, as observed in co-colonization scenarios involving *Gigaspora* [37]. Root exudates further act as a gating mechanism, with strigolactones serving as key host-derived signals that promote AMF hyphal branching and entry, while endophyte-induced alterations in exudate profiles can enhance or restrict AMF recruitment depending on colonization timing and microbial identity [38,39]. Phytohormonal prioritization provides an additional regulatory layer, as AMF colonization is closely associated with auxin and strigolactone signaling, whereas endophyte-mediated colonization frequently engages jasmonic acid- and ethylene-dependent pathways linked to induced systemic resistance. Elevated JA–ET signaling is believed to suppress AMF colonization efficiency, while balanced hormonal cross-talk permits functional complementarity between AMF nutrient acquisition and endophyte-mediated defense priming [40]. Such outcomes are regulated by MAPK–hormone signaling modules rather than passive microbial competition, suggesting a mechanistic basis for context-dependent synergism or antagonism [31,34].

Endophyte-mediated modulation also extends to microbial predators and plant parasites, including protists and nematodes. Changes in host physiology induced by endophytes can alter susceptibility to nematode invasion, either through the secretion of hydrolytic enzymes or via the activation of systemic defense pathways involving salicylic acid (SA), JA, and ET signaling [37,41]. These hormonal responses are regulated downstream of MAPK activation and NPR1-dependent signaling nodes, linking endophyte perception to defense priming across trophic levels [42]. Notably, certain endophytes are capable of colonizing nematode-induced structures such as galls, thereby modifying the internal niche environment in ways that reduce vulnerability to subsequent pathogen attack. This ability to occupy and manipulate trophic niches highlights an adaptive strategy through which endophytes influence belowground food-web dynamics [43,44]. Protist–plant interactions are similarly shaped by endophyte activity. By altering root architecture and secondary metabolite production, endophytes can reduce protist predation pressure or shift resource availability across trophic levels, thereby indirectly stabilizing microbial community structure. MAPK-regulated secondary metabolism and hormone-responsive transcription factors contribute to this trophic buffering effect [45].

Such modifications reflect an additional layer of trophic control exerted by endophytes within the rhizosphere. Among multi-trophic interactions, endophyte–pathogen relationships are the most extensively studied. Endophytes function as microbial extensions of the plant, competing with pathogens for space and nutrients and exerting direct antagonistic effects through the production of antimicrobial compounds, including lipopeptides, siderophores, and volatile organic compounds [45,46]. In parallel, endophyte-triggered MAPK signaling enhances induced systemic resistance (ISR), providing a molecular link between microbial antagonism and host immune activation [42]. Beyond direct antagonism, endophytes prime host defense systems, enabling more rapid and robust responses to pathogen invasion through ISR and systemic acquired resistance. These responses are mediated through coordinated SA–JA–ET cross-talk downstream of MPK3/MPK6 activation and transcription factors such as WRKY33 and ERF1 [31]. For example, *Phomopsis liquidambaris* was shown to reshape microbiome composition in rice spikelets, enhancing resistance to *Fusarium*-associated diseases through the enrichment of beneficial taxa such as *Pseudomonas* and increased accumulation of protective secondary metabolites [1,47]. Collectively, these findings demonstrate that endophytes operate as key regulators of multi-trophic plant–microbiome interactions, integrating microbial competition, trophic regulation, and host immune modulation to stabilize plant-associated ecosystems.

### 3.3. Immune Modulation and Host Specificity

Endophytes play an important role in fine-tuning plant immune responses while exhibiting a high degree of host specificity, enabling them to colonize plant tissues without triggering detrimental defense reactions. This immune compatibility is largely achieved through sophisticated interactions with plant pattern recognition receptors (PRRs) and the modulation of microbe-associated molecular pattern (MAMP)-triggered signaling pathways [48]. Upon PRRs activation, downstream MAPK cascades (notably MPK3 and MPK6) and calcium-dependent signaling events are selectively attenuated or reprogrammed during endophytic colonization [31,42]. Unlike pathogenic microbes that elicit strong immune responses upon MAMP perception, many endophytic taxa have evolved strategies to attenuate or bypass host immune activation. Several bacterial genera, including *Pseudomonas*, *Bacillus*, and *Azospirillum*, as well as fungal genera such as *Penicillium*, *Aspergillus*, and *Epichloë*, produce low-immunogenic or structurally modified MAMPs and secrete metabolites that interfere with receptor-mediated signaling [49,50]. These interactions may limit excessive PRR-triggered immunity while maintaining basal defense competence through MAPK–NPR1 regulatory nodes [42]. These mechanisms can suppress excessive immune activation, allowing endophytes to colonize intracellular spaces, vascular tissues, and organ-specific niches without inducing hypersensitive responses or disease symptoms [49,50]. Such immune evasion and accommodation strategies can be considered a reflection of a finely balanced host–microbe relationship rather than immune suppression. Through long-term coevolutionary processes, plants and endophytes might have established mutually beneficial and highly specific molecular interactions, wherein the host provides nutrients and habitat, while endophytes contribute to growth promotion, stress mitigation, and enhanced defense capacity [51]. Endophyte-mediated resistance is orchestrated through SA–JA–ET hormonal cross-talk integrated by transcriptional regulators such as ERF1 and WRKY33, which function downstream of MPK3/MPK6 signaling [31]. Endophyte-mediated resistance is often systemically induced via coordinated signaling through salicylic acid (SA), jasmonic acid (JA), ethylene (ET), and other phytohormones, including methyl jasmonate and brassinosteroids. This integrated hormonal network enables plants to mount rapid and effective immune responses against both biotrophic and necrotrophic pathogens [52].

Research in rice systems suggests that bacterial endophytes such as *Azospirillum* sp. B510 and various *Bacillus* strains can induce systemic resistance by activating defense-related signaling cascades. These responses include increased expression of pathogenesis-related genes (*PR1*, *PR2*, *PR3*), the upregulation of phenylpropanoid biosynthesis genes (*CHS*, *PAL*), and enhanced activity of defense-associated enzymes such as polyphenol oxidase and peroxidase compared with non-inoculated plants [53]. These transcriptional and enzymatic responses are regulated by MAPK-dependent phosphorylation events and NPR1-mediated transcriptional control [42]. Such primed immune states are further reinforced by endophyte-derived secondary metabolites, including surfactins and other lipopeptides, which directly inhibit pathogens while simultaneously acting as immune elicitors. In addition to signaling-mediated defenses, certain endophytes secrete hydrolytic enzymes such as chitinases and glucanases that degrade pathogen cell walls, thereby lowering infection pressure and modulating immune activation thresholds [1]. At the molecular level, these processes operate in parallel with MAPK–hormone signaling to prevent uncontrolled defense escalation [45]. Notably, the endophytic product ZhiNengCong (ZNC), derived from *Paecilomyces variotii* by research groups, exhibited potent immunomodulation in tobacco, highlighting the potential of endophyte-derived compounds as high-efficiency biocontrol agents [54]. Collectively, these findings underscore the capacity of endophytes to function as immune modulators and host-specific partners by integrating PRR signaling attenuation, MAPK cascade regulation, and SA–JA–ET hormonal cross-talk, enhancing resilience and productivity within complex agroecosystems. In addition to immune modulation, spatial compartmentalization further shapes endophyte function within plant microbiomes.

### 3.4. Functional Partitioning and Cross-Talk Between Endophytic and Rhizospheric Microbiomes

It has been reported since earlier times that the plant microbiome is spatially distributed into several niches, including the rhizosphere, which acts as a soil–root interface with high levels of microbial abundance and activity, and the endosphere, which consists of organisms colonizing internal plant tissues without causing disease [55,56]. Root exudates and soil physicochemical characteristics shape the rhizospheric microbial communities, which are dominated by taxa that are involved in nutrient mobilization, organic matter turnover, and the control of soil conditions that indirectly affect plant growth and health [55,57]. On the other hand, endophytic microorganisms are perceived to exhibit lower diversity and selective penetration into plant tissue that collaborates with the host’s internal microclimate and biological competency [58,59]. Comparative community studies of cotton and *Psammosilene tunicoides* have identified minimal or no mutual functional and spatial division of external and inner root-associated assemblies, with rhizospheric and endophytic microbiomes harboring few or no shared functional and spatial operational taxonomic units [58,60].

The compartmentalized functional differentiation can manifest in the prevalence of rhizospheric microorganisms, the characteristics of which are related to nutrient solubilization, siderophore production, and antagonism of soil-borne pathogens. Nonetheless, endophytes are found to be more associated with phytohormone modulation, stress tolerance, and the enhancement of host defense responses [61,62]. In spite of this compartmentalization, there continues to be widespread cross-talk between rhizospheric and endophytic microbiomes where root exudates selectively recruit microorganisms of the surrounding soil that can, in turn, colonize internal tissues, provided the right conditions exist [55,57]. Network and correlation analyses also show that certain rhizospheric taxa serve as endophyte reservoirs and are associated with host secondary metabolite accumulation, which suggests coordinated functional interactions throughout the soil root endosphere continuum [59]. Together, data presented by compartment-resolved community profiling (and functional analyses) support such a model, where rhizospheric and endophytic microbiomes are functionally segregated yet strongly interlinked by plant-mediated recruitment and metabolite-based signaling, determined by independent microbial entities [56,59].

### 3.5. Endophyte-Mediated Mitigation of Agrochemical Stress

The widespread use of agrochemicals, particularly pesticides, is associated with phytotoxicity, oxidative stress, and impaired plant metabolic functions, highlighting the need for biological strategies to mitigate these effects. Similar findings were recorded that endophytic microorganisms residing within plant tissues enhance plant resilience to toxic chemicals by mitigating oxidative stress, modulating physiological stress-response mechanisms, and facilitating the detoxification or sequestration of absorbed toxins within the host plant [63,64]. In addition, data from the rice system has shown that bacterial endophytic strains of the genus *Bacillus* found in diazinon-treated plants could use diazinon as a single carbon source and degrade this organophosphate insecticide in vitro and in the tissues of plants under laboratory conditions [65]. Similarly, consortia of naturally occurring endophytic bacteria were reported to mineralize chlorpyrifos into less toxic metabolites under controlled experimental conditions while simultaneously promoting plant growth and yield [66].

Endophytic fungal strains such as *Phomopsis* spp. have also been reported to degrade the phenoxy herbicide 4-chloro-2-methylphenoxyacetic acid with high efficiency in liquid culture and soil systems [66]. In addition to direct degradation, endophytes may alleviate agrochemical stress by enhancing antioxidant capacity, scavenging reactive oxygen species, and stabilizing cellular membranes [63,64]. For instance, studies on multiple organophosphate pesticides have recorded up to 90% degradation achieved by PGP microbes [67,68]. A review of the scientific literature suggests that endophytes may contribute to reduced reliance on chemical pesticides through enhanced stress tolerance, improved host intrinsic defense response [63]. Together, these studies support the physiological resilience of the host by balancing the adverse effects of pesticides and the like. While the present section so far focused on mechanistic and ecological principles, the following section illustrates how these processes translate into crop-specific outcomes.

## 4. Crop and Environment-Specific Applications of Endophytes

### 4.1. Abiotic Stress Response in Rice

Halotolerant endophytic bacteria inoculation, namely *Lysinibacillus fusiformis* (ART-1), *Lysinibacillus sphaericus* (ART-10), and *Brevibacterium pityocampae* (CAL-8), was associated with increased root length, shoot length, and chlorophyll content under conditions of high salinity (160 mM NaCl). Ionomic ICP-MS revealed less Na^+^ accumulation and better Mg^2+^ retention in shoots. Upregulation of OsNHX1, OsAPX1, OsPIN1, and OsCATA and downregulation of OsSOS was observed along with reduced levels of endogenous salicylic acid and abscisic acid and an increase in the level of jasmonic acid [69]. The thermotolerant bacterial endophytes *Bacillus paralicheniformis*, *B. pumilus*, and *B. paranthracis* were found to protect rice from heat stress (40–45 °C). The *B. paralicheniformis* strain inoculation resulted in 50% fresh grain weight and up to 113% dry grain weight increase was observed, along with modulation in stress response, biomolecule accumulation such as lower levels of malondialdehyde (MDA), increased proline, and salicylate and abscisic acid compared with non-inoculated controls. This suggests that these biomolecules may contribute to osmotic stress protection. Similarly, research was reported where rice was inoculated with the endophytic fungus *Piriformospora indica* at a high concentration of salt (200–300 mM NaCl), and increased chlorophyll a, chlorophyll b, and carotenoid concentrations were recorded, along with increased proline accumulation, which contributed to improved stress tolerance [70].

### 4.2. Maize Under Abiotic Stress

In maize (*Zea mays* L.), drought stress is associated with significant declines in biomass, photosynthetic performance and plant water status. Bacterial endophytes *Burkholderia phytofirmans* strain PsJN, and *Enterobacter sp*. FD17 inoculated with maize showed increases in the biomass of the shoot and roots, growth of the leaf area, and increased chlorophyll levels and photosynthesis rates relative to uninoculated plants. Additionally, the relative water content of leaves was increased by 30%, which suggests improved membrane stability [71]. Fungal endophyte *Metarhizium robertsii* exhibited enhanced growth and colonization under varying water availability. Stress-responsive levels of jasmonic acid and its conjugate, jasmonoyl-isoleucine, were observed, and it was reported that the endophyte activated the jasmonic acid biosynthetic enzyme ZmLOX1 [72].

## 5. Advances in Metagenomics and Functional Omics to Decipher Endophyte Ecology

### 5.1. Next-Generation Sequencing and Beyond

Recent advances in next-generation sequencing, particularly the maturation of long-read sequencing technologies, have substantially advanced the analysis of endophytic bacterial assemblages and plant-associated microbiomes, markedly improving taxonomic resolution, genome completeness, and functional inference [73]. Platforms developed by Pacific Biosciences and Oxford Nanopore Technologies enable contiguous sequencing reads that support near-complete or complete genome assembly, including those of rare, low-abundance, or previously uncultured endophytes. Unlike short-read sequencing approaches, which fragment genomes into small reads that are often difficult to assemble, especially in complex communities, long-read technologies overcome challenges associated with repetitive regions, high GC content, and structural genomic variation [74]. The emergence of specialized assemblers and polishers, including Shasta, Miniasm, and Flye, and long-read error-correction tools such as Racon, MarginPolish, and Medaka, has further enhanced assembly accuracy and substantially reduced base-level error rates, making high-quality metagenome-assembled genomes (MAGs) increasingly attainable [75,76].

**Figure 2:**
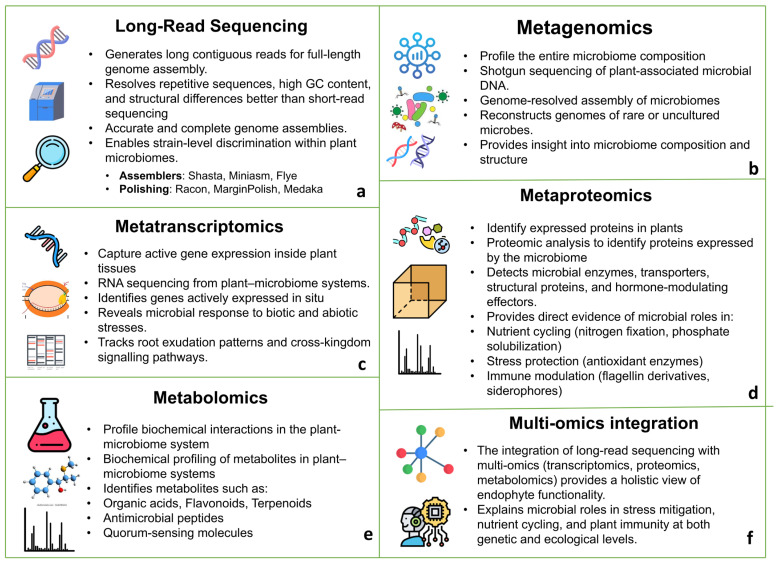
Multi-omics approaches for decoding plant–microbiome interactions and endophyte functionality. (**a**) Long-read sequencing enables high-quality genome assembly and strain-level resolution. (**b**) Metagenomics profiles microbiome composition and reconstructs genomes of plant-associated microbes. (**c**) Metatranscriptomics captures in situ gene expression and functional responses to environmental cues. (**d**) Metaproteomics identifies expressed proteins and enzymes, providing direct evidence of microbial functions. (**e**) Metabolomics characterizes biochemical interactions and signaling molecules within the plant–microbiome system. (**f**) Integration of these omics layers offers a holistic understanding of microbial roles in stress mitigation, nutrient cycling, and plant immunity.

The integration of long-read sequencing and complementary multi-omics approaches provides a comprehensive framework for understanding endophyte ecology and plant–microbiome interactions (Figure 2). A key advantage of long-read sequencing is its ability to achieve strain-level resolution, which is essential for distinguishing functionally important members of the core endophytic microbiome that may be obscured in conventional 16S rRNA gene surveys [77]. This capability is particularly critical for elucidating functional redundancy, niche specialization, and host specificity among closely related endophytic taxa. When integrated with complementary functional omics approaches, namely metatranscriptomics, metaproteomics, and metabolomics, long-read metagenomics enables a comprehensive understanding of microbial ecology that extends beyond genetic potential to realized biological function [78]. Metatranscriptomics provides insights into in situ gene expression, revealing how endophytes dynamically respond to biotic and abiotic stresses, root exudation patterns, and cross-kingdom signaling under specific environmental or host-associated conditions [79]. Metaproteomics advances this understanding by directly identifying expressed proteins, including secreted enzymes, transporters, structural proteins, and plant hormone-modulating effectors. Detection of these proteins provides evidence of microbial participation in nutrient cycling (e.g., nitrogen fixation and phosphate solubilization), stress mitigation (e.g., antioxidant enzymes), and immune modulation (e.g., siderophores and modified flagellins), often correlating closely with observed plant phenotypes [80]. In parallel, metabolomics captures the biochemical landscape generated by plant–microbe interactions, enabling the identification and quantification of metabolites such as organic acids, flavonoids, terpenoids, antimicrobial compounds, and quorum-sensing molecules. This layer of analysis offers critical insights into metabolic cross-feeding, allelopathy, signaling networks, and microbiome-driven modulation of plant physiology [81].

### 5.2. Network Biology and Microbial Interaction Mapping

Network biology and microbial interaction mapping have emerged as powerful approaches for elucidating how endophytes influence plant-associated microbiomes, particularly with respect to community structure, functional coordination, and resilience [82]. By modeling microbes as nodes and their associations as edges, network-based frameworks enable systems-level interpretation of complex plant–microbiome interactions that cannot be captured by single-taxon or abundance-based analyses alone. Co-occurrence network models, derived from microbial abundance data across multiple spatial and temporal scales, facilitate the identification of statistically significant relationships among taxa, revealing patterns of co-adaptation, mutualism, competition, or antagonism within the rhizosphere and endosphere [83]. Advanced computational tools such as SPIEC-EASI, CoNet, and SparCC address key challenges inherent to microbiome datasets, including compositionality, sparsity, and indirect correlations, thereby enabling the construction of biologically meaningful and statistically robust networks with reduced false-positive rates. Within these inferred networks, highly connected taxa (network hubs) often exhibit elevated degree and betweenness centrality, indicating disproportionate influence over information flow, resource exchange, and community stability. Such hubs—frequently represented by endophytic taxa known for nutrient cycling, immune modulation, and plant growth promotion—function as key coordinators of microbiome activity and resilience [84,85,86].

Topological features of microbial networks provide further insight into community organization. Metrics such as modularity, clustering coefficient, and assortativity reveal how microbial communities are structured and stabilized. High modularity reflects the formation of semi-independent functional units that enhance robustness against perturbations, whereas disassortative network architectures indicate the presence of highly connected generalist taxa that support specialized community members. Together, these features contribute to overall microbiome resilience in the face of environmental stressors such as drought, pathogen invasion, or agrochemical exposure [87]. Network resilience can be quantitatively assessed through simulated node-deletion analyses, which model species loss or disturbance. Robust microbial networks maintain connectivity and functional integrity even after the removal of key taxa, highlighting the importance of redundancy and compensatory interactions within endophyte-associated communities [88].

Beyond network topology, abundance–occupancy modeling offers complementary insights by distinguishing core versus transient taxa based on their frequency of occurrence and relative abundance across samples [89]. Taxa that exceed neutral model expectations—particularly those exhibiting high occupancy despite low abundance—are likely subject to deterministic host selection and represent ecologically and functionally critical members of the plant microbiome. Notably, network hubs frequently overlap with these core taxa, reinforcing their central role in maintaining both structural cohesion and functional stability within endophyte-influenced microbial networks [89,90]. Collectively, network biology provides a quantitative and predictive framework for identifying keystone endophytes, understanding emergent community properties, and guiding microbiome engineering strategies aimed at enhancing plant resilience, productivity, and sustainability.

### 5.3. Single-Cell Genomics and Spatial Metagenomics

Single-cell genomics and spatial metagenomics have significantly advanced plant microbiome research by enabling high-resolution, cell-resolved interrogation of endophytic communities. These approaches allow direct identification of metabolically active microbes, their spatial organization within host tissues, and their ecological roles across distinct plant niches [91]. In contrast, conventional bulk metagenomic methods often fail to resolve cellular heterogeneity or to reliably link functional traits to specific taxa within complex microbial consortia. Recent methodological innovations address these limitations by coupling taxonomic identity with in situ biological activity [92]. In plant systems (Arabidopsis root atlases), high-throughput single-cell RNA sequencing (scRNA-seq) has assisted in capturing precise spatiotemporal transcriptional states of individual root cell types, revealing developmental trajectories and localized gene-expression programs that are not detectable using bulk RNA-seq approaches [93]. Among these, fluorescence in situ hybridization coupled with Raman spectroscopy (Raman–FISH) enables simultaneous phylogenetic identification and functional characterization of individual microbial cells. By detecting red-shifted Raman spectra following the incorporation of stable isotopes (e.g., ^13^C or ^15^N), Raman–FISH allows direct tracing of substrate uptake and metabolic activity at the single-cell level [92,94].

Although traditionally constrained by low throughput, this limitation was overcome through stimulated Raman scattering–fluorescence in situ hybridization (SRS-FISH), which enable high-throughput phenotypic profiling of large numbers of cells within plant and environmental samples [95]. Complementary to optical methods, nanoscale secondary ion mass spectrometry (NanoSIMS) provides subcellular resolution of isotopic labeling by scanning microbial surfaces with focused ion beams (e.g., Cs^+^), enabling precise quantification of isotopic ratios such as ^15^N/^14^N, ^13^C/^12^C, and ^34^S/^33^S. This approach yields microscale maps of nutrient assimilation and metabolic fluxes within individual microbial cells [96]. When integrated with catalyzed reporter deposition–fluorescence in situ hybridization, NanoSIMS enables direct linkage of functional processes—such as nitrogen fixation or sulfur metabolism—to phylogenetically defined endophytes in situ, offering unprecedented insight into microbial activity within plant roots and associated microhabitats [97]. Spatially resolved metabolomic approaches further complement single-cell genomic analyses by enabling direct visualization of metabolite distributions within plant tissues. Another study shows an explicit relation between localized microbial colonization and metabolite redistribution across root tissues [98].

The latest technologies, such as BONCAT-FACS (bioorthogonal non-canonical amino acid tagging coupled with fluorescence-activated cell sorting), have further expanded researchers’ functional tool kit, with functional resolution enabling in situ labeling of newly synthesized proteins, which can be detected via click chemistry [99]. BONCAT-FACS, when applied to complex systems (including soil and root-associated microbiomes), revealed active protein synthesis in a substantial fraction of extractable microbial cells (~60%). Importantly, bioorthogonal non-canonical amino acid tagging (BONCAT)-labeled cells can be isolated and subjected to downstream genomic or transcriptomic analyses, thereby directly linking functional activity with genomic potential, a resolution unattainable through DNA-based sequencing alone [99,100]. These advances align with plant single-cell transcriptomic studies where cellular heterogeneity, rare cell types, and localized transcriptional responses can be resolved using scRNA-seq platforms, thereby providing a conceptual and technical foundation for detailed endophyte host interface studies [101].

The integration of single-cell genomics, isotope-enabled imaging, and spatially resolved functional assays provides a transformative framework for deciphering endophyte ecology. These approaches enable precise mapping of microbial activity, interactions, and niche specialization within plant tissues, offering critical insights into how endophytes contribute to plant health, resilience, and holobiont-level functionality. These analytical advances can further be complemented with emerging technological platforms that enable direct manipulation and optimization of endophyte traits.

## 6. Technological and Computational Innovations Shaping Endophyte Research

### 6.1. CRISPR, Genome Mining, and Metabolic Engineering

The convergence of CRISPR-mediated genome editing, genome mining, and metabolic engineering represents a promising technological triad for unlocking the biosynthetic and functional potential of endophytes in sustainable agroecosystem conditions. Together, these approaches provide a framework for the rational design of precision bioinoculants capable of conferring biocontrol activity, mitigating environmental stress, and enhancing crop productivity; however, their practical deployment remains constrained by regulatory approval processes, ecological risk considerations, strain stability, and challenges associated with consistent colonization and performance under field conditions [102,103]. Endophytic bacterial and fungal genomes frequently harbor abundant biosynthetic gene clusters (BGCs) encoding diverse secondary metabolites, including antimicrobials, antioxidants, siderophores, and phytohormones. However, a substantial proportion of these BGCs remain cryptic or transcriptionally silent under standard laboratory conditions, necessitating advanced genome mining and epigenetic activation strategies to access their functional potential [104,105]. Contemporary antibiotic and metabolite discovery pipelines increasingly rely on high-throughput in silico platforms such as antiSMASH and PRISM to identify, annotate, and predict the chemical outputs of BGCs. These predictions are further validated through integrated transcriptomic, proteomic, and metabolomic analyses, enabling temporal mapping of BGC expression and metabolite production [106,107].

The activation of silent BGCs was achieved by scientists using epigenetic elicitors, including DNA methyltransferase inhibitors (e.g., 5-azacytidine) and histone deacetylase inhibitors (e.g., SAHA and valproic acid), leading to the discovery of novel bioactive compounds with agricultural and pharmaceutical relevance [108,109]. Complementing these approaches, CRISPR/Cas9 genome editing has emerged as a powerful and precise tool for functional manipulation of endophyte genomes, enabling the targeted deletion, insertion, or modification of BGCs and stress-responsive genes. In endophytic fungi such as *Epichloë* spp., CRISPR-based ribonucleoprotein complexes were employed to non-transgenically eliminate genes involved in toxic alkaloid biosynthesis (e.g., *dmaW* and *lolC*), thereby generating forage-safe symbionts without compromising beneficial host interaction [110,111]. Similarly, in *Pestalotiopsis fici*, CRISPR/Cas9-mediated genome editing achieved high-efficiency targeted insertions (48%) and dual-locus mutations (44.4%), substantially reducing the number of transformation steps required in conventional Agrobacterium-based systems [112]. In *Phomopsis liquidambaris*, CRISPR-mediated knockout of a mitogen-activated protein kinase kinase gene resulted in enhanced biosynthesis of pharmaceutically valuable flavonoids such as kaempferol and quercetin, demonstrating the feasibility of pathway redirection through targeted editing [12].

Beyond pathway activation, genome editing has enabled metabolic engineering for the overproduction of phytohormones (e.g., indole-3-acetic acid), antifungal metabolites, and nutrient-acquisition enzymes, thereby improving plant tolerance to drought, salinity, and oxidative stress [113]. For instance, engineered *Pseudomonas* strains showed overproduction of zeaxanthin diglucoside (an antioxidant) and turnerbactin (a siderophore), highlighting the feasibility of developing multi-trait endophytes capable of simultaneously promoting growth and stress resilience [114]. To translate these engineered strains into effective field applications, significant advances have been made in bioformulation and delivery systems. Microbial immobilization strategies using alginate- or pectin-based hydrogels protect endophytes from environmental stress while enabling controlled, sustained release. For example, encapsulated *Kosakonia radicincitans* increased radish tuber dry matter by 18.9% and leaf dry matter by 20.7% under field conditions [115]. In parallel, nano-enabled formulations, including natural charcoal-based nanoparticles, have demonstrated synergistic disease suppression and enhanced defense gene activation in *Rhizoctonia solani*-challenged rice systems [116]. Bioformulations employing organic carriers such as compost and biochar further improve endophyte viability, facilitate integration into native soil microbiomes, and enhance nutrient cycling and plant immune competence [117]. Collectively, the integration of genome mining, CRISPR-based editing, metabolic engineering, and advanced formulation technologies provides a potential framework for converting endophytic microbes into next-generation, climate-resilient bioinputs for sustainable and regenerative agriculture.

### 6.2. AI/ML and Bioinformatics Platforms

Plant microbiome research is increasingly leveraging AI, machine learning (ML), and advanced bioinformatics to enable predictive microbiome engineering and to establish mechanistic links between microbial community features and plant phenotypes [118]. These computational approaches are essential for analyzing the high-dimensional and complex datasets generated by next-generation sequencing and multi-omics platforms, thereby facilitating a deeper understanding of how microbial communities collectively influence plant growth, stress tolerance, and disease resistance [119]. A wide range of supervised and unsupervised ML algorithms, including decision trees, support vector machines, random forests, and neural networks, are now routinely applied to uncover weak, nonlinear, and multivariate relationships between microbial abundance patterns, functional gene expression, and plant phenotypic traits [120]. Such models outperform traditional statistical methods in capturing emergent properties of microbiomes, particularly under heterogeneous environmental conditions. High-resolution taxonomic and functional profiling of plant-associated microbiomes across the rhizosphere, endosphere, and phyllosphere is enabled by computational pipelines such as QIIME2, PICRUSt2, MetaPhlAn, and HUMAnN3 [50,121]. These tools allow comprehensive integration of taxonomic composition with functional potential, forming the foundation for downstream predictive modeling.

Concurrently, curated databases and statistical frameworks linking microbiome features to host phenotypes now support genome-wide and systems-level association studies. These resources enable the identification of specific microbial taxa, functional genes, or metabolic pathways associated with plant traits such as root architecture, nutrient acquisition efficiency, flowering time, and resistance to biotic and abiotic stresses [14,122]. Such associations are critical for moving from correlative observations to hypothesis-driven microbiome manipulation. Advanced computational platforms further enable microbial network modeling, allowing the identification of keystone taxa and interaction modules that govern ecosystem functioning and microbiome stability under changing environmental conditions [123]. AI-assisted simulations and network-based analyses can help predict the behavior of synthetic microbial consortia, including colonization success, metabolic cooperation, competitive exclusion, and functional redundancy, prior to field deployment [14,124,125]. These predictive capabilities may reduce trial-and-error approaches traditionally associated with microbial formulation development and enhance host compatibility by emulating realistic plant–microbiome–environment interactions [126].

**Figure 3:**
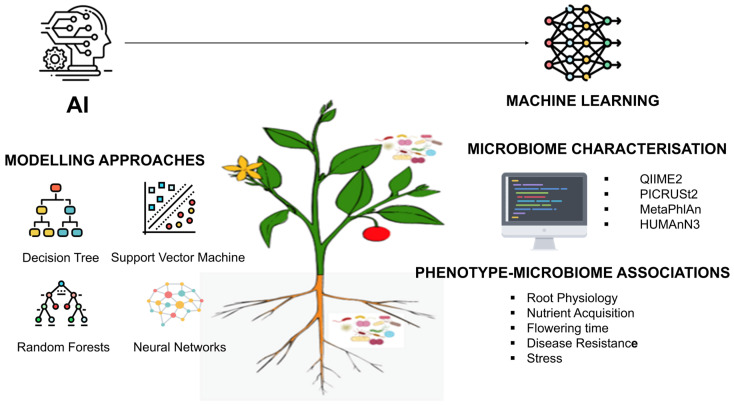
Conceptual overview of artificial intelligence (AI) and machine learning (ML) applications in plant–microbiome research. The framework depicts computational modelling approaches (decision trees, support vector machines, random forests, and neural networks), microbiome characterization tools (QIIME2, PICRUSt2, MetaPhlAn, HUMAnN3), and the integration of microbial, genomic, and phenotypic data to infer associations between microbiome features and plant traits, including root physiology, nutrient acquisition, flowering time, disease resistance, and stress tolerance.

Deep learning architectures, particularly convolutional neural networks and recurrent neural networks, are increasingly applied to microbiome datasets for tasks such as microbial classification, source tracking, and functional gene prediction [127]. These models excel at learning hierarchical patterns from large datasets and are especially suited for integrating heterogeneous data layers, including genomics, transcriptomics, metabolomics, and phenotypic traits [124]. AI- and ML-based frameworks are increasingly used to analyze complex plant–microbiome datasets and predict plant performance outcomes (Figure 3). Despite these developments, there are several constraints that reduce the usefulness and applicability of predictive models. The domain shift that occurs between tightly controlled laboratory data and field-specific heterogeneity may significantly reduce the predictive power of in silico-trained models [128]. The heterogeneity of environmental conditions, a variation in the genotype of the host, soil non-homogeneity, and the existence of unmeasured ecological confounders may add bias and predictive stability across different locations and seasons [129]. Further, microbiome data have a compositional nature, are frequently sparse, and lie in a high-dimensional format, environments that can create spurious correlations or overfitting unless special care is taken to ensure that rigorously determined normalization, transformation, and validation steps are used. The above considerations highlight the need to carefully cross-environment validate as well as interpret AI-/ML-generated predictions before any translational implementation [130].

Collectively, AI-/ML-driven bioinformatics platforms are transforming endophyte research from descriptive microbiome profiling toward predictive, design-oriented microbiome engineering, accelerating the development of targeted, climate-resilient, and crop-specific biological solutions. Despite these technological breakthroughs, several translational limitations remain that constrain field-level application.

## 7. Limitations and Challenges in Translating Endophyte Research to Field Applications

Although significant advances were made in deciphering the benefits of conferred endophytes inoculation under controlled conditions, the extrapolation of such results to large-scale agronomic systems is still limited, owing to vast variations in biological and ecological conditions [131,132]. One of the most common challenges is the instability of colonization, where the introduced microbes often fail in sustaining consistent population numbers in open field settings. This shortcoming is attributed to a lack of appropriate niches, poor competitive ability, and antagonistic action with resident soil microbiota [133,134]. These instabilities are conferred due to spatial and temporal heterogeneity in field soil due to variations in the environment [135]. The impact of these intrinsic factors can be seen in the fact that, in some cases, the positive outcome observed in a greenhouse or growth chamber environment may drastically decline or may disappear in divergent agroecological conditions. The significance of host genotypes and pre-existing microbial networks, as the prominent driver of microbial associations, cannot be ruled out. Thus, it is not a surprise that PGP traits in single-strain inoculation studies may fail to manifest within complex native microbiomes [131,132].

A major challenge limiting the large-scale use of endophyte-based technology in the agriculture sector is regulatory and biosafety concerns. The intentional introduction of microbial inoculants raises significant concerns about unintended ecological impacts, non-target interactions, and long-term consequences on native microbial diversity and ecosystem stability [133]. More importantly, endophyte lifestyles are not dichotomous, as they might pass a spectrum ranging between mutualism and pathogenicity based on host genotype and environmental conditions, thus complicating biosafety assessments and the regulatory approval pathway [131,136]. Problems of scalability, formulation, and reproducibility still present significant challenges to the popularization of endophyte-based strategies. Many variables play a decisive role in using endophytes in agronomic settings, depending not only on the rationality of strains but also on the physicochemical stability of formulations, the selection of carrier matrices, appropriate storage regimes, and the mechanism of delivery [132,133]. An overview of these limitations highlights the need to adopt integrative methods that consider environmental heterogeneity, microbial community dynamics, host-specific interactions, and regulatory considerations rather than pitching one’s conclusion by near-extrapolation of findings from experiments carried out under controlled conditions (in vitro; greenhouse) [131,132]. These challenges frame the broader implications and future directions of endophytic research.

## 8. Conclusions

Endophytes are now gaining traction as a key mediator of plant–microbiome interactions, influencing plant health, stress tolerance, and ecosystem processes through coordinated biochemical and ecological mechanisms. Findings from multi-omics, systems biology, and single-cell approaches indicate that endophytes may restructure microbial communities, modulate plant signaling pathways, and contribute to resilience under abiotic stress. The incorporation of genome mining, metabolic engineering, and AI-driven analytics further enables data-guided identification and design of promising strains and synthetic consortia tailored to specific crops and environments. Moving toward field validation requires several concrete priorities. A key priority is the development of standardized and reproducible multi-omics pipelines, including harmonized sampling, data processing, and cross-platform validation. Another requirement is the establishment of clear criteria for defining optimal endophytes, incorporating functional efficacy, colonization stability, host compatibility, and biosafety considerations. Additionally, a staged validation framework progressing from controlled greenhouse conditions to multi-location field trials is essential to assess performance under realistic environmental variability. Cumulatively, rigorous validation and responsible deployment are likely to be critical for translating mechanistic insights into consistent agricultural outcomes. Experimentally validated development of endophyte-based interventions may contribute to resilient cropping systems, reduced chemical inputs, and improved soil functionality under changing climatic conditions.

## Data Availability

No new data were created or analyzed in this study.

## Abbreviations

The following abbreviations are used in this manuscript:

AI	Artificial intelligence
AMF	Arbuscular mycorrhizal fungi
BGCs	Biosynthetic Gene Clusters
BONCAT	Biorthogonal non-canonical amino acid tagging
BONCAT-FACS	Biorthogonal non-canonical amino acid tagging combined with fluorescence-activated cell sorting
CRISPR	Clustered regularly interspaced short palindromic repeats
ET	Ethylene
ISR	Induced systemic resistance
JA	Jasmonic acid
MAMPs	Microbe-associated molecular patterns
ML	Machine learning
PGP	Plant growth-promoting
PRRs	Pattern recognition receptors
SA	Salicylic acid
